# Numerical Modeling of the Dynamic Elastic Modulus of Concrete

**DOI:** 10.3390/ma16113955

**Published:** 2023-05-25

**Authors:** Gustavo de Miranda Saleme Gidrão, Ricardo Carrazedo, Rúbia Mara Bosse, Laura Silvestro, Rodrigo Ribeiro, Carlos Francisco Pecapedra de Souza

**Affiliations:** 1 Department of Civil Engineering, Federal University of Technology-Paraná (UTFPR), Guarapuava 85053-525, PR, Brazil; rubiambosse@utfpr.edu.br (R.M.B.); laurasilvestro@utfpr.edu.br (L.S.); rodrigosribeiro@utfpr.edu.br (R.R.); pecapedra@utfpr.edu.br (C.F.P.d.S.); 2 School of Engineering of São Carlos, University of Sao Paulo, Av. Trabalhador Saocarlense, 400, Sao Carlos 13566-590, SP, Brazil

**Keywords:** dynamical properties, dynamic elastic modulus, concrete, mortar, mixture parameters, acoustic test, composite theory

## Abstract

This article introduces simulations of theoretical material with controlled properties for the evaluation of the effect of key parameters, as volumetric fractions, elastic properties of each phase and transition zone on the effective dynamic elastic modulus. The accuracy level of classical homogenization models was checked regarding the prediction of dynamic elastic modulus. Numerical simulations were performed with the finite element method for evaluations of the natural frequencies and their correlation with *E_d_* through frequency equations. An acoustic test validated the numerical results and obtained the elastic modulus of concretes and mortars at 0.3, 0.5 and 0.7 water–cement ratios. Hirsch calibrated according to the numerical simulation (x = 0.27) exhibited a realistic behavior for concretes of w/c = 0.3 and 0.5, with a 5% error. However, when the water-to-cement ratio (w/c) was set to 0.7, Young’s modulus displayed a resemblance to the Reuss model, akin to the simulated theoretical triphasic materials, considering matrix, coarse aggregate and a transition zone. Hashin-Shtrikman bounds is not perfectly applied to theoretical biphasic materials under dynamic situations.

## 1. Introduction

Dynamic modulus of elasticity (*E_d_*) is related to a very small instantaneous strain, geometrically similar to the initial tangent modulus [1]. Mehta and Monteiro [1] reported E_d_ higher than the initial tangent modulus in approximately 20%, 30% and 40%, for concretes of high, medium and low resistance. Nevertheless, the relation between the static and dynamic moduli is not precise, since no physics law correlates their values to the necessary precision [1,2,3]. The use of *E_d_*, which can be accurately determined by acoustic tests [4,5], is more appropriate for analyses of structures subjected to impact loading [1], e.g., vibration serviceability limit state.

*E_d_* is strongly influenced by mixture parameters, such as the water–cement ratio of mortar (porosity), maturity, the elastic properties of coarse aggregate, the volumetric fraction of the interfacial transition zone (ITZ) and the aggregate–cement proportion [1,3,6,7,8].

Classical models predict with success the elastic modulus of a perfect biphasic composite. Since the 19th century, homogenization models, such as Voigt (or parallel) [1,9,10,11,12] and Reuss [1,10,11,12,13] (or series), have been used to determine the elastic static properties of concretes. According to Hill [14], Voigt and Reuss are upper and lower bounds for any biphasic material. Hashin-Shtrikman (H-S) [11,15] developed the most stringent bounds of behavior for a composite material [11,15,16,17]. Nilsen and Monteiro [16,17] showed limitations in the application of H-S bounds, since concrete is not a perfectly biphasic material. Consequently, a third composite component (i.e., transition zone, ITZ) must be considered in the model. In 2013, Nemat-Nasser and Srivastava [18] reported another limitation of H-S bounds: the approach cannot apply to dynamic situations perfectly. This is a severe limitation, especially for the estimation of dynamic elastic modulus through modal analysis.

Other classical homogenization models have predicted the elastic properties of biphasic materials. Counto [1,11] assumed a prismatic coarse aggregate in the center of the mortar prism, whereas Hansen [1,11] adopted a spherical coarse aggregate in the same region. Hirsch [1,11] proposed a semi empirical model that relates the modulus of elasticity of the concrete to the Young’s modulus’ of the two phases (aggregate and matrix), their volume fractions and an empirical constant, x. The “x value” is a combination factor obtained by the Voigt and Reuss models and calibrated either experimentally, or via a numerical simulation. Topçu [11] obtained x = 0.3 experimentally. The present study aims to obtain x through numerical simulations.

Sophisticated models have been proposed towards predictions of effects, such as the size and shape of aggregate particles and the influence of porosity and transition zone [19,20,21,22,23,24,25,26]. Hashin and Monteiro [27] described concrete as a three-phase material formed by a matrix with embedded spherical particles and surrounded by a concentric spherical shell that represents an ITZ. According to the authors, the Young’s modulus of ITZ is 50% of the elastic modulus of the cement mortar (i.e., *E*_ITZ_ = *E_m_*/2). Duplan et al. [28] designed a very realistic model constituted by aggregates surrounded by a layer of ITZ and a layer of cement paste, while air bubbles are considered mono-sized inclusions with no elastic behavior, which result in a 5% error. Zheng, Zhou and Jin [22,23] proposed a model in which concrete is represented as a three-phase composite material composed of mortar, spherical or elliptical aggregate and an inhomogeneous ITZ, based on a semi-empirical gradient model, dependent on the water–cement ratio, the degree of hydration and the porosity at the ITZ. Pichler et al. [26] predict the dynamic modulus of cement pastes with w/c = 0.35 and 0.60, considering a material constituted by clinker, pores (with spherical shapes) and hydration products (with spherical and/or non-spherical shapes).

Although such models are complex for practical applications, those recent studies have opened up new possibilities for the micromechanical modeling of concrete through numerical simulations, especially simulations that allow the study of dynamic elastic modulus (*E_d_*). The current research focuses on simulations of the concrete as a 2D theoretical material (biphasic or triphasic) with controlled elastic properties of each phase (mortar and aggregate). The purpose is to evaluate the behavior of dynamic effective elastic modulus (*E_d_*) regarding porosity, individual elastic properties of coarse aggregate and/or mortar (i.e., *E_a_*, *ν_a_*, *E_m_* and *ν_m_*, respectively), volumetric fraction and the presence of ITZ with known properties (i.e., ITZ thickness and elastic modulus *E*_ITZ_). Further, this approach with controlled properties was compared with classical models, such as Voigt, Reuss, Hirsch, H-S, Hansen and Counto, aiming to show the most accurate classical model for predicting the *E_d_*. An experimental validation showed the coherence and limitations of our numerical simulations. Therefore, this paper advances the methodology to predict the dynamic elastic modulus of concrete and contributes to the comprehension of its response under dynamical excitations.

## 2. Numerical Simulations

**Figure 1:**
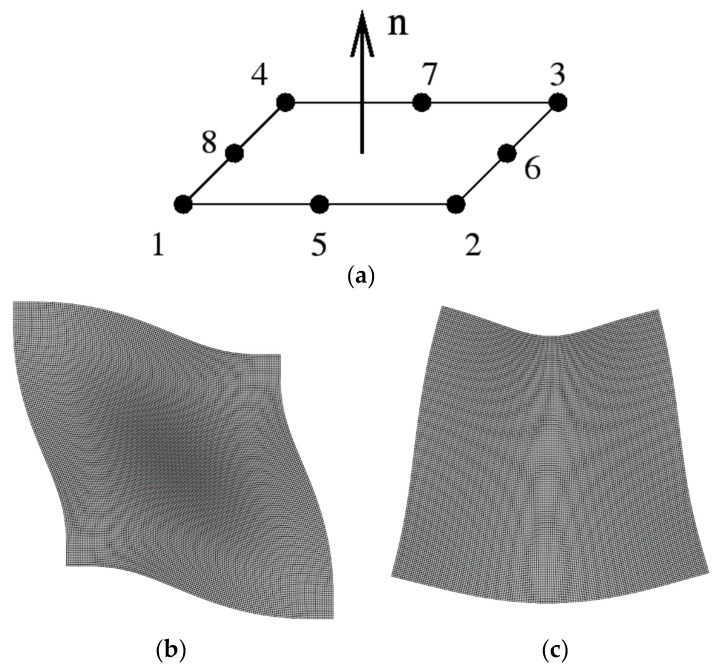
Vibration modes considered in the numerical analysis. (**a**) CPS4R element with 8 integration nodes; (**b**) “Shear” mode; (**c**) “Bending” mode.

Numerical simulations were performed with the general-purpose finite element software Abaqus release 6.14. A theoretical material was modeled by a 150 mm × 150 mm × 50 mm square with fined mesh 1 mm maximum dimension and a quadrilateral and four-node bilinear element with reduced integration (CPS4R, Figure 1a). Plane stress was considered in this 2D shell structure. This model was chosen due to the low computational cost. The boundary conditions were not considered in the model, representing a free model subject to vibrations.

In this study, modal analysis in Abaqus with the Lanczos solution method was utilized to investigate the dynamic behavior of a free shell structure. The Lanczos solution is an iterative method that enables the calculation of the eigenvectors and eigenvalues of a large sparse symmetric matrix, making it ideal for modal analysis. The shell structure was modeled using a linear elastic material with a Poisson ratio and elastic modulus, and a density of 2400 kg/m^3^ was considered. The shell was allowed to vibrate freely to demonstrate all modes of vibration. Abaqus was employed to generate the mass and rigidity matrices and to determine the eigenvalues and eigenvectors for the shell structure.

### 2.1. Frequency Equations of an Isotropic and Homogeneous Material

The relationship between the first natural frequency (bending or shear mode of vibration) and effective elastic properties are established. The theoretical modal analysis determines the shear and flexural first natural frequency for a free vibration of a homogeneous isotropic square structure (see Figure 1). “Shear” frequency can be related to the Effective Dynamic Shear Modulus (*G_d_*), whereas “flexural” frequency is associated with Effective Dynamic Elastic Modulus (*E_d_*) [4,5,29] through “Frequency equations”.

**Figure 2:**
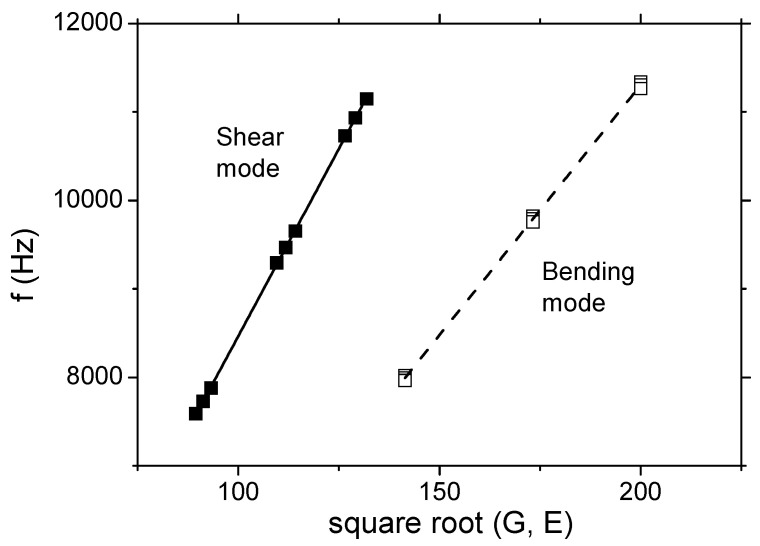
Vibration modes considered in the numerical analysis.

Table 1 shows an analysis performed with a homogeneous isotropic material of *ρ* = 2400 kg/m^3^ density and variations in the elastic and shear modulus. Simulations determined the frequency equation and confirmed the expected relationships of the “shear” frequency and the “shear” modulus (Equation (1)), as well as the “bending” frequency and the elastic modulus (Equation (2)), see Figure 2. Constants *C*_1_ and *C*_2_ are dependent on the geometry of the specimens and were determined by a linear regression analysis, *C*_1_ = 131.16 and *C*_2_ = 87.56 (with R^2^ → 1), respectively.

(1) $$f_{shear}=C_{1}\sqrt{\frac{G_{d}}{\rho }}$$

(2) $$f_{bend}=C_{2}\sqrt{\frac{E_{d}}{\rho }}$$

### 2.2. Effect of Porosity

**Figure 3:**
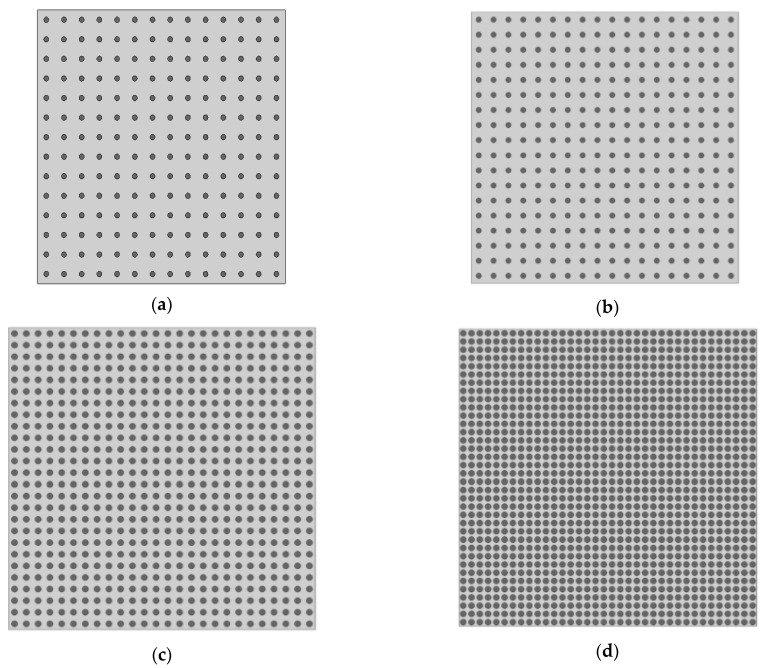
Models of porosity simulation. (**a**) 169 voids resulting in 5.31% porosity; (**b**) 324 voids resulting in 10.18% porosity; (**c**) 676 voids resulting in 21.24% porosity; (**d**) 1296 voids resulting in 40.7% porosity.

**Figure 4:**
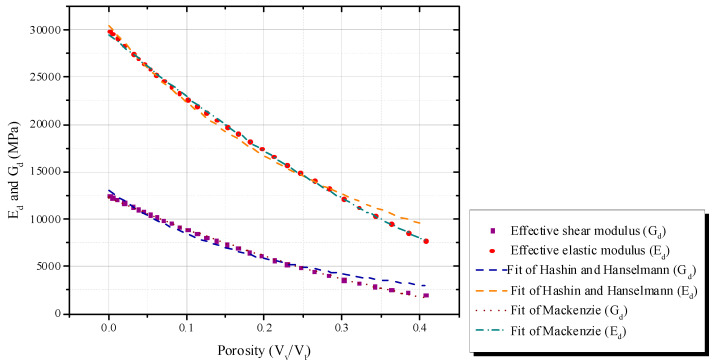
Numerical correlation of *E_d_* or *G_d_* and porosity.

The influence of porosity (P) was checked on effective elastic properties *G_d_* and *E_d_*. We suppose a theoretical material constituted by spherical voids of 3 mm diameter, distributed in a matrix (e.g., Figure 3). Voids were incrementally added to the matrix, i.e., four voids (2 by 2–0.13% porosity) until 1296 voids (36 by 36–40.72% porosity). For all cases, the cement paste had constant properties of *ρ_p_* = 2100 kg/m^3^, *E_p_* = 20,000 MPa and *ν_p_* = 0.2, whereas the voids had *ρ_v_* = 1 kg/m^3^ and *E_v_* = 0,1 MPa (i.e., *ρ_v_/ρ_p_* = 0.0004 and *E_v_*/*E_p_* = 0.000005). An approximately 0.13% to 40.72% porosity increase decreased *E_d_* (Figure 4), since porosity is inversely linked to the material rigidity [8]. Boccacini and Fan [30] reviewed several theoretical models to correlate *E_d_* and the porosity of ceramic materials. Hanselmann-Hashin [31,32] (Equation (3)) and Mackenzie [33] (Equation (4)) achieved the best fit with numerical results (see Figure 4 and Table 2). The fit with R^2^ close to 0.97 showed coherence of simulations.

(3) $$E=E_{0}\left(1+\frac{AP}{1-(A+1)P}\right)$$

(4) $$E(P)=E_{0}[1-K\times P+(K-1)\times P^{2}]$$

where *E* is the effective elastic modulus, *E*_0_ is the intact elastic modulus under porosity equal to zero (value recovered from *E*_0_ ≅ 30,000 MPa), *P* is porosity and *A* and *K* are empirical adjustments.

### 2.3. Biphasic Material

A perfectly biphasic material with coarse aggregate (of elastic modulus *E_a_*) perfectly encrusted in a mortar phase (with elastic modulus *E_m_*) without transition zone was simulated and the influence of individual elastic properties of each phase (*E_a_*/*E_m_*), volumetric fraction of the aggregates and Poisson ratio of each phase on effective *E_d_* was checked. The accuracy of homogenization composite models was measured under the above-mentioned conditions.

#### 2.3.1. Influence of Individual Elastic Properties of Phases (*E_a_*/*E_m_*)

**Figure 5:**
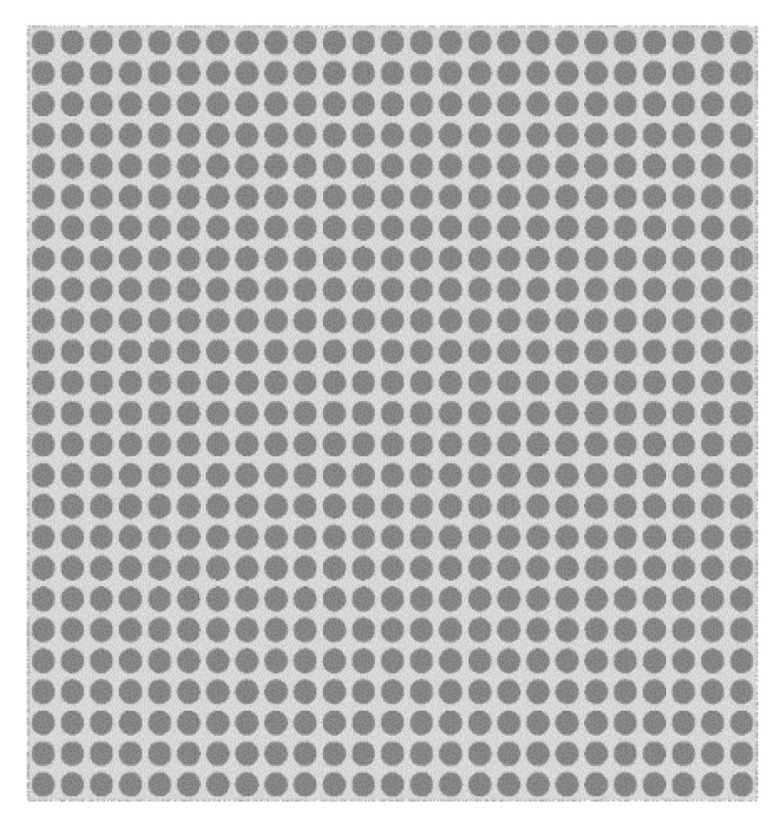
Matrix with encrusted aggregates.

We supposed an effective material composed of 625 perfectly spherical aggregates of 4.8 mm diameter, arranged in a mortar, with no transition zone (ITZ) and/or porosity, as shown in Figure 5. An analysis was performed with mortar properties of *ρ_m_* = 2100 kg/m^3^, *E_m_* = 10,000 MPa, 20,000 MPa or 30,000 MPa and *ν_m_* = 0.2, and aggregate properties of *ρ_a_* = 2700 kg/m^3^, *E_a_* = 30,000 MPa, 60,000 MPa or 90,000 MPa and *ν_a_* = 0.2.

**Figure 6:**
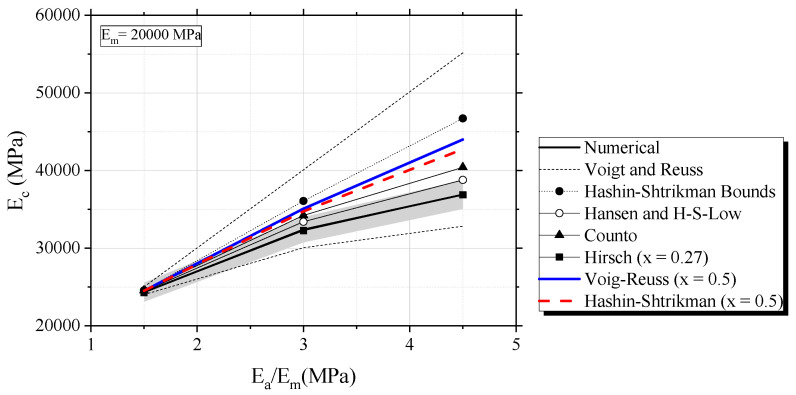
Evaluation of biphasic estimates and influence of the elastic modulus of the aggregate on effective *E_d_*.

Figure 6 shows an increase in *E_a_* increases *E_d_* in the same volumetric proportion and constant *E_m_*, explained by the “Mixture Rule” [9,13]. The gray area in Figure 6 represents a ± 5% error, as of the effective model. This error is used in the verification of the accuracy of classical models and is similar to the admissible error adopted by Duplan et al. [28]. When the elastic modulus of the mortar (*E_m_* = 20,000 MPa) and aggregates (*E_a_* = 30,000 MPa) were relatively close (i.e., *E_a_*/*E_m_* = 1.5), the prediction of *E_d_* for all classical models was accurate; however, when *E_a_/E_m_* > 2, almost all homogenization models overestimated effective *E_d_* (i.e., Voigt, H-S, Counto, mean of H-S and Voigt-Reuss). An exception to this was the Reuss model (series), which underestimated *E_d_* for all cases of *E_a_/E_m_*. The Hirsch model calibrated to x = 0.27 accurately represented the phenomenon. The x value is very similar to that obtained experimentally by Topçu for static elastic modulus [11] (i.e., x = 0.3). H-S-Low (and Hansen) also showed a good approximation level, with an almost 5% error. The data indicates that the H-S bounds include the effective biphasic material, while the estimation of the H-S lower bound demonstrates good accuracy in representing the increase of Ea/Em. According to Nilsen and Monteiro [16], the H-S limits diverge because concrete is not considered a biphasic material and a third phase (i.e., transition zone) should be included. This is partially true, since a portion of error due to biphasic H-S does not consider the ITZ phase; nevertheless, Figure 6 shows only cases of a perfectly biphasic material with no ITZ. This divergence of H-S and the biphasic material occurs because the Hashin-Shtrikman bounds were formulated from a static consideration [18], which is not valid here. Therefore, H-S bounds are not perfectly applied to dynamic situations and should be used with caution for modal analysis. Voigt and Reuss bounds always contain a biphasic material, and a simple average of those limits was inferior to 10%, when *E_a_/E_m_* < 3. The same tendency was observed for the H-S average. On the other hand, when *E_a_* extrapolates *E_a_/E_m_* > 3, Voigt-Reuss and H-S significantly diverge, which causes a very high error.

**Figure 7:**
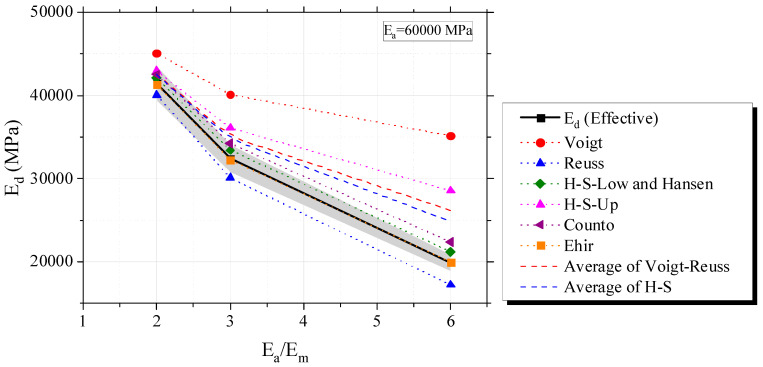
Evaluation of biphasic estimates and influence of the elastic modulus of the mortar on *E_d_*.

In the same volumetric proportions and *E_a_*, a decrease in *E_m_* (and increase in ration *E_a_/E_m_*) decreases *E_d_*, according to Figure 7. Classical biphasic models presented the same anteriorly postulated tendencies.

#### 2.3.2. Effect of Volumetric Fraction

**Figure 8:**
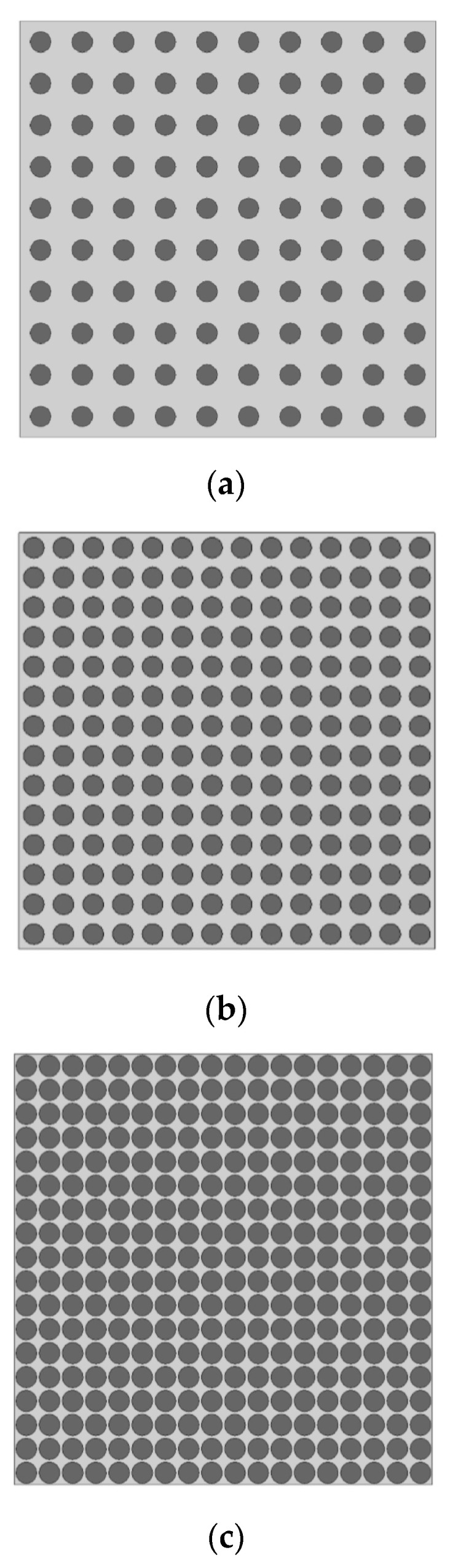
Models used in the evaluation of volumetric variation. (**a**) 196 particles—19.63% of aggregate; (**b**) 196 particles—38.48% of aggregate; (**c**) 324 particles—63.62% of aggregate.

Variations in the volumetric portion can also influence *E_d_*, with fixed *E_a_* and *E_m_* [1,3,34]. Theoretical materials whose volumetric fraction increases progressively (i.e., 196 to 324 particles, according to Figure 8) were simulated. The aggregate diameter, elastic modulus and density of each phase were constant (ϕ_a_ = 7.5 mm, *E_a_* = 90,000 MPa, *E_m_* = 20,000 MPa, *ρ*_a_ = 2700 kg/m^3^ and *ρ*_m_ = 2100 kg/m^3^).

**Figure 9:**
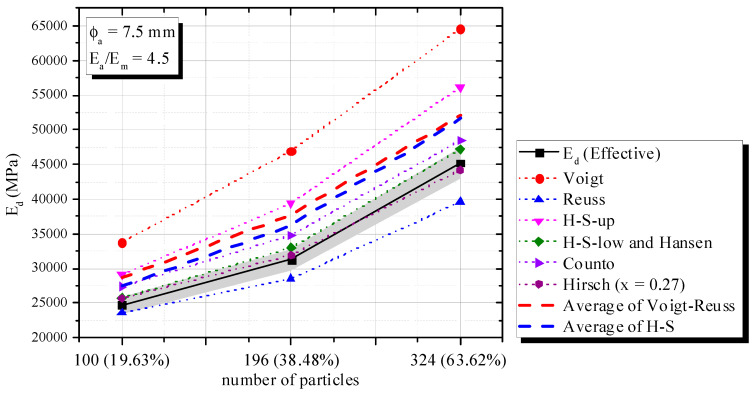
Influence of number of particles and volumetric fraction on *E_d_*.

Figure 9 shows the correlation between volumetric fraction and *E_d_*. In this case, an H-S and/or Voigt–Reuss average did not describe the phenomenon successfully, since *E_a_/E_m_* > 3. Hirsch x = 0.27 and H-S-Low accurately captured the phenomenon with a 5% error. All tendencies already mentioned are observed.

### 2.4. Triphasic Composite Material

The models were constituted by coarse aggregate, mortar and a transition zone with known properties, i.e., 0.05 mm [35,36]–0.25 mm thickness and *E_m_*/2 (*E*_ITZ_ = 10 GPa) elastic modulus [1,27,35,36]. Two diameters of aggregates, namely ϕ_a_ = 4.8 mm (256 particles) and ϕ_a_ = 7.5 mm (625 particles), were used at 50.27% constant volumetric fraction (c_a_). For simplicity and reductions in the computational costs, the theoretical material contains no voids.

**Figure 10:**
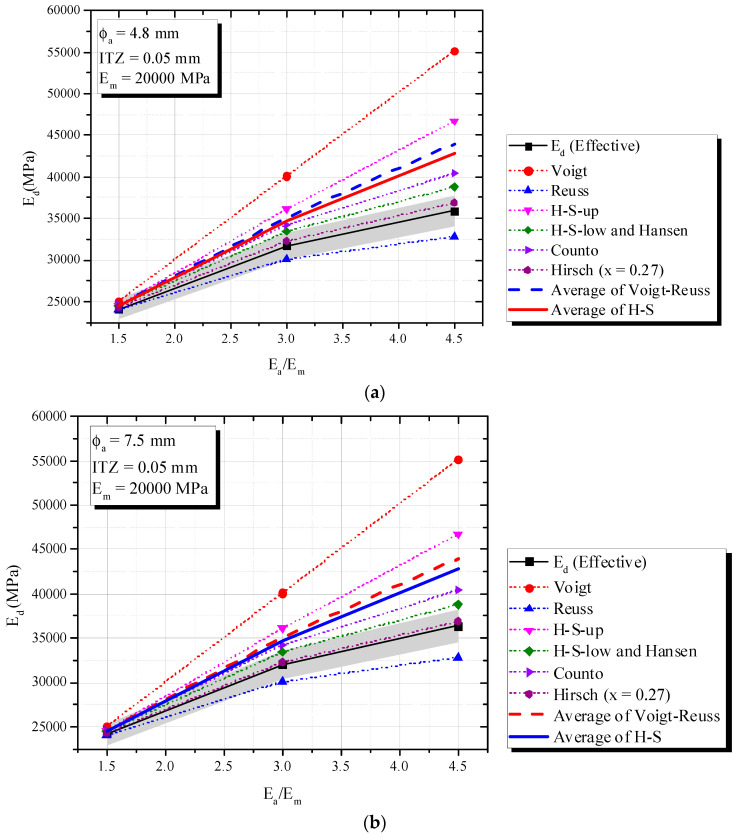
Influence of *E_a_/E_m_* (triphasic composite with ITZ = 0.05 mm) on *E_d_* in comparison with biphasic models. (**a**) ϕ_a_ = 4.8 mm and ITZ = 0.05 mm.; (**b**) ϕ_a_ = 7.5 mm and ITZ = 0.05 mm.

Figure 10 (ITZ of 0.05 mm), 15 (ITZ of 0.10 mm) and 16 (ITZ of 0.25 mm) show an increase in *E_a_/E_m_* increased the composite elastic modulus (*E_d_*), fixed mortar-aggregate proportions and *E_m_* = 20,000 MPa. This tendency is very similar to that observed in simulations of a theoretical biphasic material.

**Figure 11:**
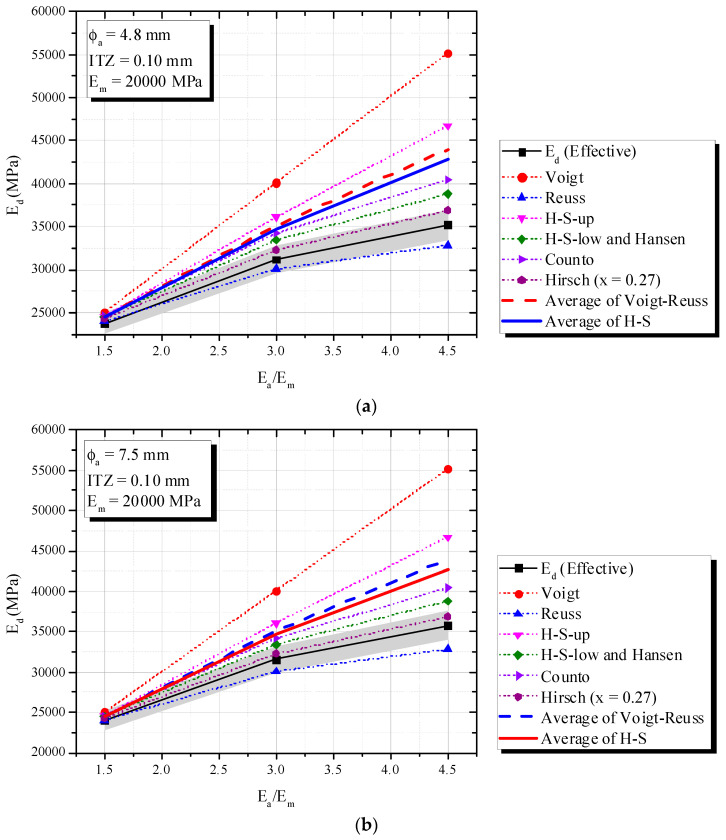
Influence of *E_a_/E_m_* (triphasic composite with ITZ = 0.10 mm) on *E_d_* in comparison with biphasic models. (**a**) ϕ_a_ = 4.8 mm and ITZ = 0.10 mm; (**b**) ϕ_a_ = 7.5 mm and ITZ = 0.10 mm.

**Figure 12:**
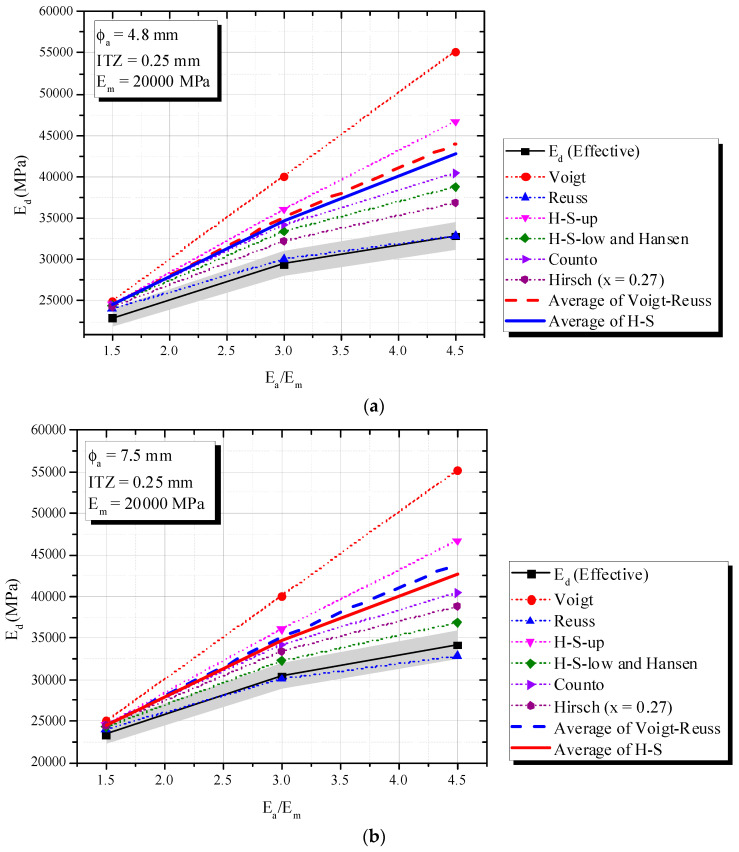
Influence of *E_a_/E_m_* (triphasic composite with ITZ = 0.25 mm) on *E_d_* in comparison with biphasic models. (**a**) ϕ_a_ = 4.8 mm and ITZ = 0.25 mm; (**b**) ϕ_a_ = 7.5 mm and ITZ = 0.25 mm.

According to Figure 10 and Figure 11, the Hirsch model with x = 0.27 best predicted *E_d_* for ITZ of 0.05 mm and 0.10 mm, with a 5% error. However, it failed to capture the phenomenon when ITZ increased to 0.25 mm and the behavior of the triphasic model was similar to the inferior biphasic model, i.e., Reuss (see Figure 12).

Hashin-Shtrikman confirmed again its limitations, i.e., the influence of ITZ and the application of H-S under dynamic situations. Although H-S bounds showed limitations, H-S-Low revealed good accuracy when ITZ was 0.05 mm (Figure 10) and *E_a_/E_m_* < 3. A simple average of Voigt–Reuss or H-S showed errors higher than 5%.

**Figure 13:**
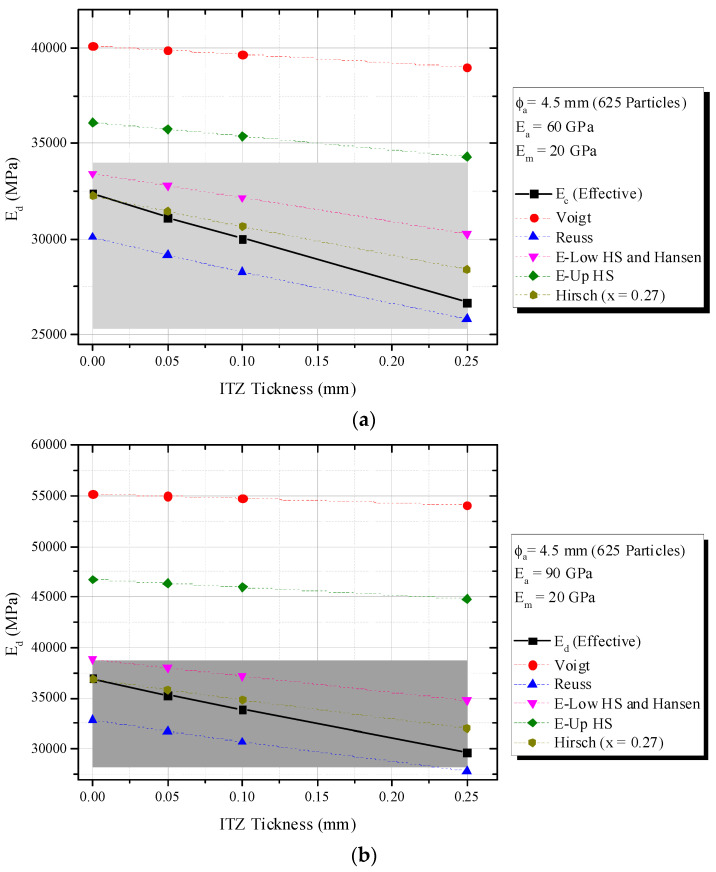
Triphasic model prediction and influence of ITZ thickness. (**a**) ϕ_a_ = 4.5 mm, *E_a_/E_m_* = 3 and variable ITZ; (**b**) ϕ_a_ = 4.5 mm, *E_a_/E_m_* = 4.5 and variable ITZ.

Classic biphasic models can consider ITZ, if applied twice, for the obtaining of a triphasic material. The methodology consists in the obtaining of a preliminary composite constituted by mortar and ITZ and associated with a coarse aggregate for the generation of a triphasic composite. Figure 13 shows the numerical data for ϕ_a_ = 4.5 mm, *E_a_/E_m_* = 4.5 or 3, *E_m_* = 20,000 MPa and variable ITZ. Although ITZ is incorporated, the same tendencies above mentioned are observed.

## 3. Experimental Validation

### 3.1. Materials and Mixes

**Figure 14:**
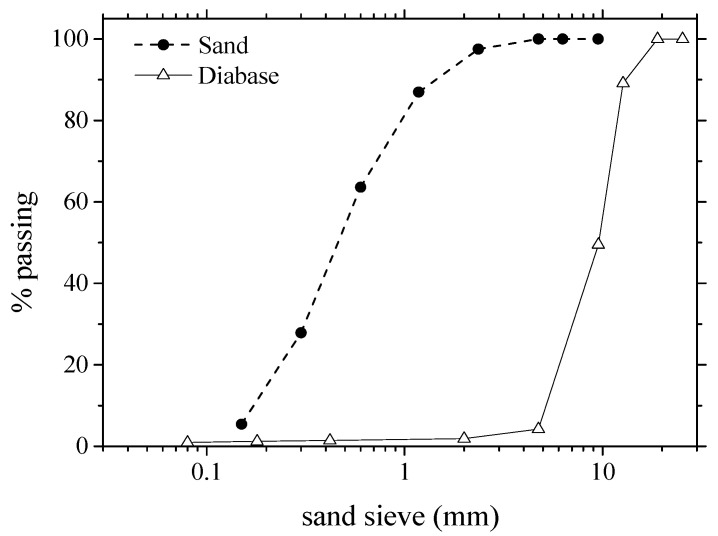
Grading curves of aggregates.

Concrete and mortar samples were produced for the validation of the numerical study. Portland cement type III of 3.10 g/cm^3^ density, specified according to the classification of ASTM C150 [37] and ABNT NBR 5733:1991 [38], was used in the samples. Natural sand of 2.19 fineness modulus [39] and 2.65 g/cm^3^ density and crushed diabase of 3.30 g/cm^3^ density and 12.5 mm nominal maximum sizes were used as aggregates. Their grading curves are shown in Figure 14.

The proportions (in weight) of mortar and concrete mixtures are shown in Table 3. Water cement ratios, amounts of sand, coarse aggregates and superplasticizers were varied among the mixtures. The production of mortars and/or concrete samples consisted in (i) mix the cement and aggregates with no water addition, then (ii) water was added and the mixing procedure was performed until the material had shown a homogeneous appearance. After hardening, the molds were removed and immediately stored in a moist chamber for curing until the age of testing.

### 3.2. Specimens

The following samples were produced:

(i) Mortars (1-M, 2-M and 3-M)—five prismatic specimens of 40 mm × 40 mm × 160 mm.
(ii) Concretes (1-C, 2-C and 3-C)—five prismatic specimens of 150 mm × 150 mm × 500 mm.
(iii) Rocks’ samples—five cylindrical samples of 55 mm × 125 mm created by core drills extracted from an intact diabase rock.

### 3.3. Acoustic Tests and Evaluation of Experimental E_d_

**Figure 15:**
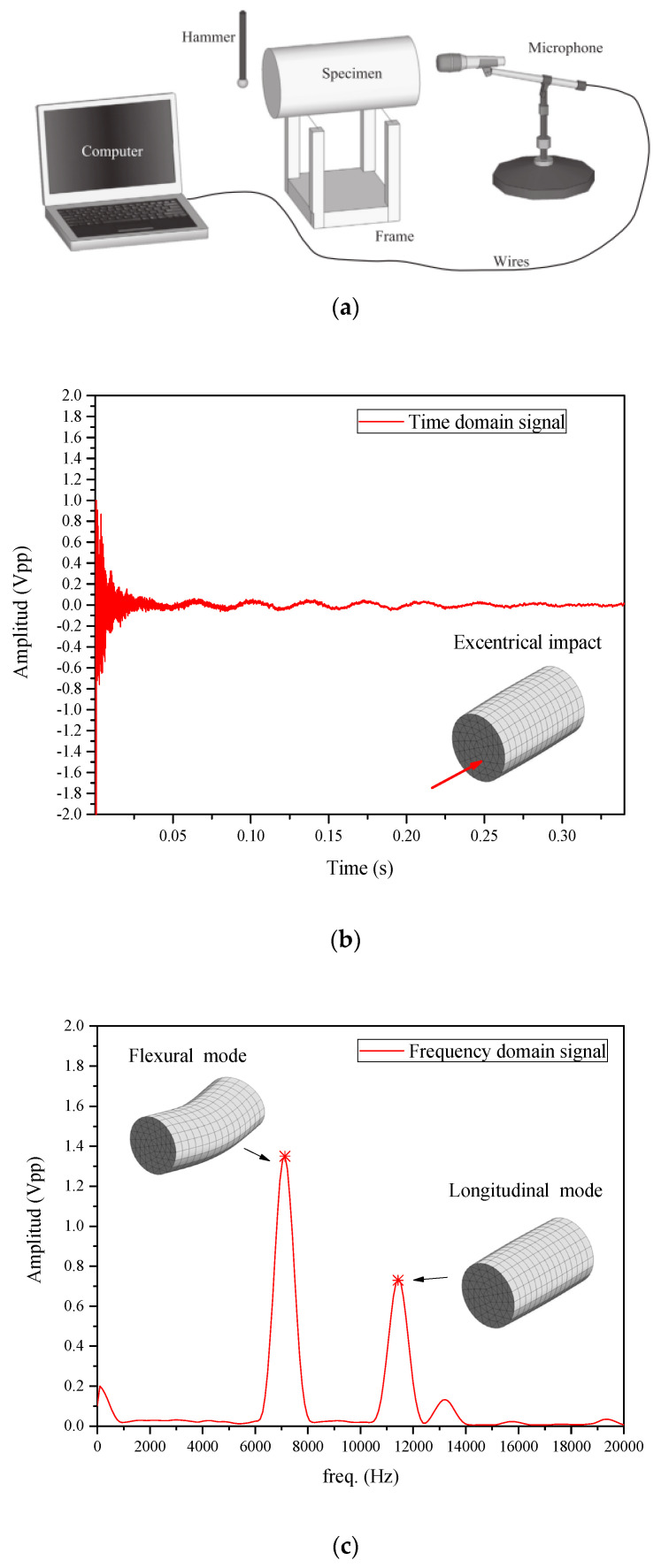
(**a**) Scheme of acoustic test; (**b**) time domain; and (**c**) frequency domain.

**Figure 16:**
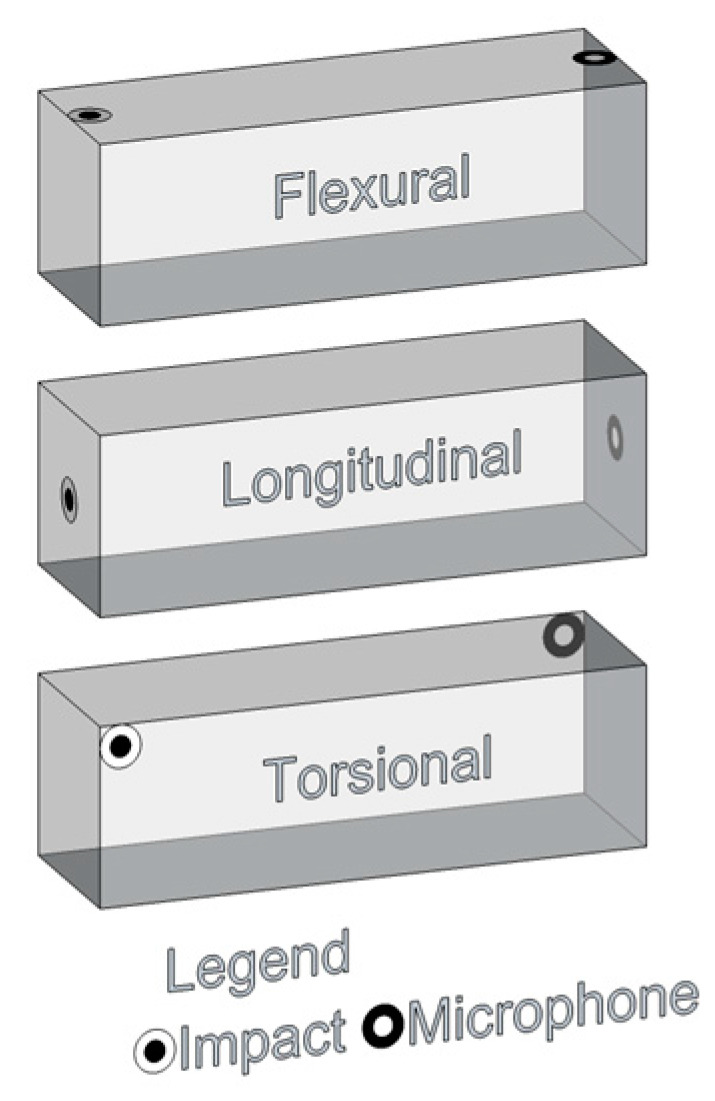
Excitation and measurement positions for the different vibration modes investigated.

**Figure 17:**
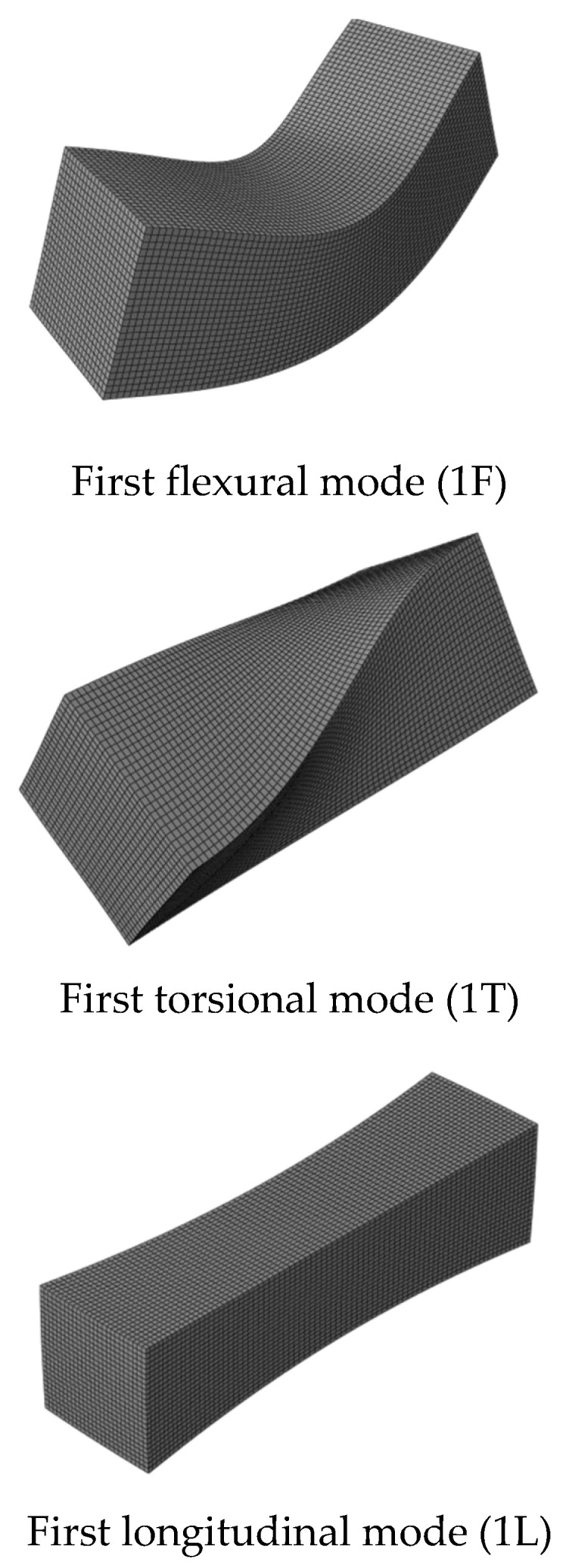
Fundamental vibration modes investigated.

Acoustic tests (Figure 15a) were performed in mortars and concrete specimens, according to ASTM C215 [4]. The mass of each sample was measured prior to each acoustic test. The sample was then positioned on steel wires attached to a metal frame (Figure 15a) and an impact was manually applied with a hammer on the surface of the specimen, while a microphone captured the sound radiated by the specimen’s surface in another position. Depending on the vibration mode evaluated (flexural, torsional or longitudinal), different impact and measurement positions were selected, as suggested by ASTM E1876-01 [5] and shown schematically in Figure 16. The first two modes of each type (flexural, torsional and longitudinal) (see Figure 17) were easily identified in the prismatic specimens. However, this study focused on 1st flexural and 1st longitudinal modes (see Figure 15c and Figure 17), i.e., the necessary vibration modes for the obtaining of the dynamic elastic modulus (*E_d_*). After excitation, an onboard sound card of a regular notebook captured the acoustic signal at a 96 kHz acquisition rate. Figure 15b shows a typical time vs. amplitude signal in a prismatic concrete sample tested at 28 days of age, obtained from an impact applied for activating the flexural and longitudinal modes (eccentric excitation). The first 1024-point block was selected, multiplied by a Hanning window and then zero-padded for the obtaining of an 8192-point vector. A fast Fourier transform was applied for the detection of peaks of natural frequencies.

Once experimental *f*_1,*flex*_ and *f*_1,*long*_ are obtained by acoustic test and exciting flexural and/or longitudinal vibrations modes, the elastic properties are evaluated through frequency equations, according to ASTM E1876 [5]. The sample’s dynamic modulus of elasticity (*E_d_*) was obtained from the first flexural mode of vibration (*E_d,f_*) using Equations (5) and (6) for prismatic or cylindrical specimens, respectively:

(5) $$E_{d,f}=0.9465\frac{mL^{3}}{bt^{3}}f_{flex,1}{}^{2}B$$

(6) $$E_{d,f}=1.6067\frac{mL^{3}}{D^{4}}f_{f}{}^{2}A$$

where *b* and *t* are dimensions of the prismatic cross section, *D* is the diameter of the cylindrical cross section, *L* is the specimen length, m is the specimen mass (in kg) and *A* and *B* are a correction factor dependent on the Poisson coefficient (*ν*, generally 0.20, according to Mehta and Monteiro [1]) and dimensions of the specimens (*b*, *t* and *D*), computed by Equations (7) and (8):

(7) $$A=1+4.939(1+0.0752\upsilon_{d}+0.8109\upsilon_{d}{}^{2}){\left(\frac{D}{L}\right)}^{2}-0.4883{\left(\frac{D}{L}\right)}^{4}-\left[\frac{4.691\left(1+0.2023\upsilon_{d}+2.173\upsilon_{d}{}^{2}\right){\left(\frac{D}{L}\right)}^{4}}{1.000+4.754\left(1+0.1408\upsilon_{d}+1.536\upsilon_{d}{}^{2}\right){\left(\frac{D}{L}\right)}^{2}}\right]$$

(8) $$B=1+6.585(1+0.0752\upsilon_{d}+0.8109\upsilon_{d}{}^{2}){\left(\frac{t}{L}\right)}^{2}-0.868{\left(\frac{t}{L}\right)}^{4}-\left[\frac{8.340\left(1+0.2023\upsilon_{d}+2.173\upsilon_{d}{}^{2}\right){\left(\frac{t}{L}\right)}^{4}}{1.000+6.338\left(1+0.1408\upsilon_{d}+1.536\upsilon_{d}{}^{2}\right){\left(\frac{t}{L}\right)}^{2}}\right]$$

Similarly, the dynamic modulus of elasticity was obtained from the first longitudinal mode of vibration (*E_d,l_*), in accordance with Equation (9) for prismatic specimens:

(9) $$E_{d,l}=4\frac{mL}{bt}f_{l}{}^{2}\frac{1}{K}$$

In this case, *K* is a correction factor given by Equation (10) and *D_e_* is the effective diameter of the bar, equal to *D* for a cylinder and given by Equation (11) for a prismatic specimen.

(10) $$K=1-\frac{\pi^{2}\nu_{d}{}^{2}D_{e}{}^{2}}{8L^{2}}$$

(11) $$D_{e}{}^{2}=2\frac{b^{2}+t^{2}}{3}$$

### 3.4. Experimental Results and Discussion

The problem of evolution of elastic modulus over time was chosen to demonstrate the observed tendencies presented in the above numerical results. All curves were calibrated by CEB FIB [40] (Equations (12) and (13)) for ages of 1 to 60.

(12) $$E(t)=\beta_{E}\times E_{28}$$

where *E*(*t*) is the function that represents the evolution of the elastic modulus over time, *E*_28_ is the modulus of elasticity at 28 days and *β_E_* is the contribution of time, according to Equation (13):

(13) $$\beta_{E}={\left\{\exp\left\{s\left[1-{\left(\frac{28}{t}\right)}^{0.5}\right]\right\}\right\}}^{0.5}$$

**Figure 18:**
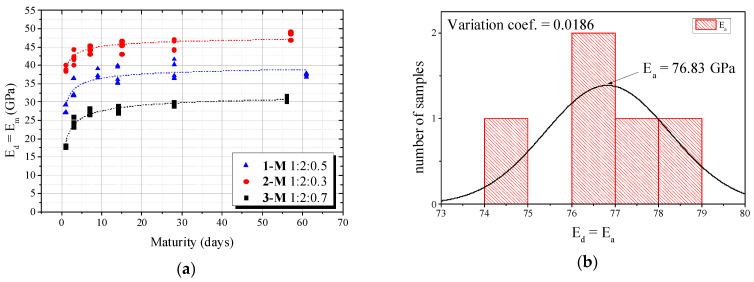
*E_d_* for (**a**) mortars and (**b**) diabasic rock.

The input of the biphasic model was the evolution over time of the dynamic elastic modulus of mortar *E_m_* (see Figure 18a) and the dynamic elastic modulus of samples of diabase rock *E_a_* (see Figure 18b).

**Figure 19:**
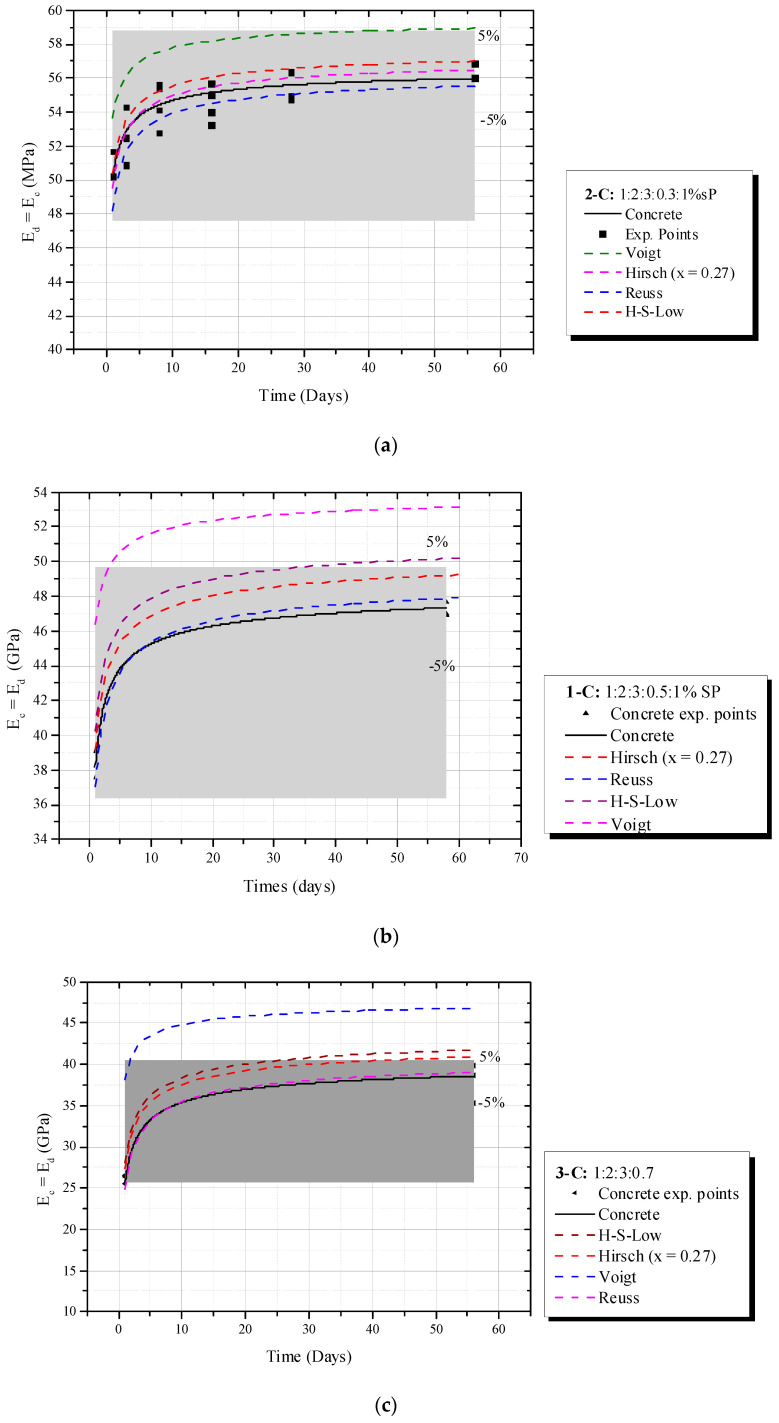
Evolution of *E_d_* for w/c = (**a**) 0.3, (**b**) 0.5 and (**c**) 0.7.

Figure 19a–c shows the behavior of the dynamic elastic modulus of the concrete (*E_d_*) over time and the biphasic models that predicted this evolution.

The experimental investigation showed Hirsch (x = 0.27) achieved good accuracy when w/c = 0.3 and 0.5, with errors within the 5% limit and identical to the maximum error observed in numeric simulations. However, its absolute error was slightly higher than 5% for concretes with w/c = 0.7. This phenomenon is explained by a complex interaction of mortar, ITZ, water and coarse aggregate surface at early ages. According to Simeonov and Ahmad [17], a fresh concrete mixture undergoes a process of water migration to the surface of the coarse aggregates and this “water attraction” increases the ITZ thickness. Such a decrease is more pronounced at high water–cement ratios (e.g., w/c = 0.7); the *E_d_*(*t*) curve moves to the Reuss model, which also justified the same tendency of concretes with w/c = 0.5 (within 5% error). Therefore, numerical simulations revealed that, when ITZ increases, the best prediction is achieved by the Reuss model, which is similar to the experimental behavior. On the other hand, this phenomenon is reduced for concretes with little ITZ (e.g., w/c = 0.3) and dynamic elastic modulus is very close to Hirsch (x = 0.27).

## 4. Conclusions and Final Remarks

The research involved the development of numerical simulations for a porous material that is biphasic and triphasic, with a focus on investigating the dynamic elastic modulus. The key findings of the study are:

(i) Homogenization composite models such as Reuss and Hirsch (i.e., x = 0 and x = 0.27) provide good accuracy to predict the dynamic elastic modulus, presenting a maximum error of 5%. Hirsch (x = 0.27) is the most reliable predictor of *E_d_* for ITZ values of 0.05 mm and 0.10 mm, while the Reuss model is the best fit for ITZ = 0.25 mm. In experimental studies, a similar trend was observed, where Hirsch (x = 0.27) successfully predicted the *E_d_*(*t*) phenomenon for concretes with low and moderate water–cement ratios (w/c = 0.3 and 0.5), while the Reuss was the only model to predict the mixture with w/c = 0.70 with an error consistently lower than 5%.
(ii) In both numerical simulations and experimental validation, Voigt, H-S, and Hansen models consistently produce overestimated values for *E_d_*.
(iii) Hashin-Shtrikman limits do not contain biphasic theoretical models and cannot be applied to dynamic situations perfectly [18].
(iv) The effective Poisson ratio determined by means of natural frequency measurements demonstrates a notable margin of error, owing to the anisotropy produced by the configuration and orientation of coarse aggregates within the mortar. Furthermore, the arrangement of these coarse aggregates may also affect the measurement, as varying arrangements with equivalent elastic properties and volumetric fractions can result in divergent Poisson ratio values.

The present study has improved our comprehension of how concrete responds to linear dynamic excitations. Through numerical simulations of the theoretical material, the classical models have been refined and evaluated.

## Figures and Tables

**Table 1:** Elastic parameters and corresponding frequencies.

*E* (MPa)	*ν*	*G* (MPa)	$\sqrt{E}$	*f_bend_* (Hz)	$\sqrt{G}$	*f_shear_* (Hz)
20,000	0.15	8696	141.4	8020	93.3	7881
20,000	0.2	8333	141.4	7994	91.3	7730
20,000	0.25	8000	141.4	7965	89.4	7587
30,000	0.15	13,043	173.2	9822	114.2	9652
30,000	0.2	12,500	173.2	9790	111.8	9467
30,000	0.25	12,000	173.2	9756	109.5	9292
40,000	0.15	17,391	200.0	11,341	131.9	11,145
40,000	0.2	16,667	200.0	11,305	129.1	10,931
40,000	0.25	16,000	200.0	11,265	126.5	10,729

**Table 2:** Porosity data.

Model	Parameter	*K* or *A*	*E*_0_ or *G*_0_ (MPa)	R^2^
Mackenzie	G	*K* = 2.896	*G*_0_ = 12,340.899	0.99872
Mackenzie	E	*K* = 2.370	*E*_0_ = 29,593.245	0.99935
Hanselmann-Hashin	G	*A* = −4.976	*G*_0_ = 13,148.495	0.97948
Hanselmann-Hashin	E	*A* = −3.314	*E*_0_ = 30,590.435	0.99187

**Table 3:** Unitary mix proportions of mortars and concretes.

ID	Cement (kg)	Sand (kg)		Water (kg)	Superplasticizer (kg)
1-M	1.00	2.00		0.50	0.010 (1%)
2-M	1.00	2.00		0.30	0.010 (1%)
3-M	1.00	2.00		0.70	0
**ID**	**Cement** **(kg)**	**Sand** **(kg)**	**Coarse Aggregate** **(kg)**	**Water** **(kg)**	**Superplasticizer** **(kg)**
1-C	1.00	2.00	3.00	0.50	0.010 (1%)
2-C	1.00	2.00	3.00	0.30	0.010 (1%)
3-C	1.00	2.00	3.00	0.70	0

## Data Availability

Not applicable.
